# Carbohydrates in Human Milk and Body Composition of Term Infants during the First 12 Months of Lactation

**DOI:** 10.3390/nu11071472

**Published:** 2019-06-28

**Authors:** Zoya Gridneva, Alethea Rea, Wan Jun Tie, Ching Tat Lai, Sambavi Kugananthan, Leigh C. Ward, Kevin Murray, Peter E. Hartmann, Donna T. Geddes

**Affiliations:** 1 School of Molecular Sciences, M310, The University of Western Australia, Crawley, Perth, WA 6009, Australia; 2 Centre for Applied Statistics, The University of Western Australia, Crawley, Perth, WA 6009, Australia; 3 School of Human Sciences, The University of Western Australia, Crawley, Perth, WA 6009, Australia; 4 School of Chemistry and Molecular Biosciences, The University of Queensland, St. Lucia, Brisbane, QLD 4072, Australia; 5 School of Population and Global Health, The University of Western Australia, Crawley, Perth, WA 6009, Australia

**Keywords:** human milk carbohydrates, lactose, oligosaccharides, infant, body composition, lactation, daily intake, breastfeeding frequency, milk intake

## Abstract

Human milk (HM) carbohydrates may affect infant appetite regulation, breastfeeding patterns, and body composition (BC). We investigated relationships between concentrations/calculated daily intakes (CDI) of HM carbohydrates in first year postpartum and maternal/term infant BC, as well as breastfeeding parameters. BC of dyads (*n* = 20) was determined at 2, 5, 9, and/or 12 months postpartum using ultrasound skinfolds (infants) and bioelectrical impedance spectroscopy (infants/mothers). Breastfeeding frequency, 24-h milk intake and total carbohydrates (TCH) and lactose were measured to calculate HM oligosaccharides (HMO) concentration and CDI of carbohydrates. Statistical analysis used linear regression/mixed effects models; results were adjusted for multiple comparisons. Higher TCH concentrations were associated with greater infant length, weight, fat-free mass (FFM), and FFM index (FFMI), and decreased fat mass (FM), FM index (FMI), %FM and FM/FFM ratio. Higher HMO concentrations were associated with greater infant FFM and FFMI, and decreased FMI, %FM, and FM/FFM ratio. Higher TCH CDI were associated with greater FM, FMI, %FM, and FM/FFM ratio, and decreased infant FFMI. Higher lactose CDI were associated with greater FM, FMI, %FM, and FM/FFM, ratio and decreased FFMI. Concentrations and intakes of HM carbohydrates differentially influence development of infant BC in the first 12 months postpartum, and may potentially influence risk of later obesity via modulation of BC.

## 1. Introduction

Nutrition [1] and development of infant body composition (BC) in the early months postpartum [2] are known to play a significant role in the programming of obesity. Breastfeeding and its duration are associated with reduced risk of developing obesity later in life [3]. These observations suggest a dose-dependent effect of breastfeeding on the development of infant BC, but the mechanisms of this effect are not fully elucidated. Although it is well established that added dietary sugar is a risk factor for obesity for children and adults [4] as well as infants [5] and contributes to metabolic diseases [6], little is known how human milk (HM) carbohydrates influence the development of BC during infancy and if they confer protection against the obesity.

The macronutrient composition of HM is remarkably conserved across populations appearing to be largely independent of geographic and ethnic factors [7], maternal nutritional status [8], and adiposity [9]. HM provides a constant supply of carbohydrates to the infant during early life, ensuring appropriate nutrition, maturation and development of their comparatively immature physiological systems. Considering that lactose, the principal carbohydrate, is important for maintenance of constant osmotic pressure in HM [10], it is unlikely that maternal adiposity would have a significant impact on lactose concentration, which is estimated to be approximately 60–78 g/L throughout the lactation, although lactose concentration is still variable between women [11]. As lactose synthesis results in water being drawn into the milk, the rate of lactose synthesis is a major controlling factor of milk production [12], and during the established lactation higher lactose concentration is associated with higher 24-h volumes [13] and higher breastfeeding frequency [14], which is also related to higher 24-h volumes [15]. 

HM carbohydrates also include small amounts of monosaccharides, such as glucose and galactose, as well as HM oligosaccharides (HMO). Nearly 200 unique oligosaccharides structures that vary from 2 to 22 sugars have been identified [16] and HMO composition and proportion varies between mothers [17], over the course of lactation [18] and is affected by parity, ethnicity, and place of residence, as well as breastfeeding exclusivity [19], and may exert different effects in the infant gut [20]. This suggests that HM carbohydrates may have a dose-dependent effect on infant growth and BC development.

Infant growth rate is associated with the volume of milk consumed [11] and lactose/carbohydrate intakes are lower in breastfed infants at 3 and 6 months compared to formula-fed infants and positively relate to weight and fat-free mass (FFM) but not fat mass (FM) gain [21]. Contradictory, studies of lactose concentration have reported either positive association of HM lactose with infant adiposity at 12 months [22] or no association with infant BC at 6 months [23]. Furthermore, there are differing associations for different HMO (16 HMO investigated) with infant BC, although total concentration was not analyzed [20]. In addition, concentrations of glucose are positively associated with relative weight and both fat and lean mass of breastfed infants [24]. The investigations in this area are extremely limited and need to discern the mechanisms by which HM carbohydrates, concentrations and intakes, may impact development of infant BC, allowing for future targeted interventions that may reduce risk of overweight and obesity later in life. 

The purpose of this pilot longitudinal study was to assess the associations of concentration and daily intake of HM carbohydrates (total carbohydrates (TCH), lactose, and HMO) with anthropometric measurements and BC of healthy term infants breastfed on demand and their mothers during the first 12 months of lactation. Relationships of HM carbohydrates with infant 24-h milk intake and breastfeeding frequency were also investigated.

## 2. Materials and Methods

### 2.1. Study Overview

We curried out a longitudinal cohort study of mothers with milk supply within a normal range [25], and healthy term infants that were exclusively breastfed [26] at 2 and 5 months and continued breastfeeding on demand until 12 months postpartum without supplementation with formula. The study design, cohort characteristics, data collection procedures, and sample preparation have been described in detail previously, together with the methods for determining maternal and infant anthropometrics and BC [15,27,28], as well as infant 24-h milk intake [29] and breastfeeding frequency [25], and the relationships between the measured parameters.

Briefly, milk samples were collected and BC of healthy dyads (no suspected infectious illness, e.g., a cold or flu, at the time of study visit) was determined when the infants were two and/or five, nine, and 12 months-old utilising bioelectrical impedance spectroscopy (BIS; infants and mothers), and ultrasound for measurements of the skinfolds (ultrasound 2-skinfolds: triceps and subscapular; ultrasound 4-skinfolds: biceps, subscapular, suprailiac, and triceps; infants only) for use in the age and sex-specific equations [15,30]. Infant test-weighing before and after every breastfeeding from each breast over 24–26 h was conducted to measure infant 24-h milk intake and breastfeeding frequency between 2 and 5 months, when milk intake has been reported to be stable [27], and within two weeks of 9 and 12 months. Mothers also estimated and self-reported breastfeeding frequency at the study sessions as the current typical time (h) between the meals. 

In addition to standard measured BC parameters (fat-free mass (FFM), fat mass (FM), and percentage FM (%FM)) we have calculated the height-normalized BC indices of mothers and infants: FFM index (FFMI) was calculated as FFM/length^2^; FM index (FMI) was calculated as FM/length^2^, both expressed as kg/m^2^ [31]. The use of these BC indices is considered less statistically flawed than %FM [32], which includes a single variable (FM) in both numerator and denominator. Furthermore, the dyads’ FM to FFM ratios (FM/FFM), which are emerging as values indicative of overall effects of BC (metabolic load and metabolic capacity) [33] were also calculated.

All mothers provided written informed consent for participation in the study, which was approved by the Human Research Ethics Committee of The University of Western Australia (RA/1/4253, RA/4/1/2639) and registered with the Australian New Zealand Clinical Trials Registry (ACTRN12616000368437). 

### 2.2. Measurement of Human Milk Carbohydrates

Lactose concentration was measured in duplicate in pre- and postfeed samples using the enzymatic-spectrophotometric method [11] with recovery of 98.2 ± 4.1% (*n* = 10), detection limit of 30 mM and interassay coefficient of variation (CV) of 3.5%, and averaged for analysis.

Pre- and postfeed samples were pooled for measuring TCH. Skim HM was deproteinized with trichloroacetic acid [34] before dehydration by sulfuric acid [35]. TCH were analyzed in triplicate by UV spectrophotometry at 315nm. Recovery of added lactose was 101.4% ± 2.1% (*n* = 7) with a detection limit of 0.007 g/L and an interassay CV of 3.3% (*n* = 7).

The total HMO concentration (g/L) was estimated by deducting lactose concentration from TCH concentration. On two occasions, where the calculated HMO concentration was less than zero, it was taken to be zero. Glucose and galactose were not measured or accounted for in HMO concentration calculations, as their concentrations in HM are small and thus not detectable in these assays [36].

### 2.3. Calculated Daily Intakes of Carbohydrates

24-h milk intakes values measured during 24-h test-weighing, and HM carbohydrate concentrations measured in samples taken at the study sessions at corresponding time point were used for calculation of infant daily intakes (CDI). As there are no significant circadian [11] and short-term (weekly) [37] variations in HM carbohydrates concentration during the established lactation, these CDI were considered representative of a typical daily intake.

### 2.4. Statistical Analyses

This longitudinal study, the details of which, along with power calculation have been described previously [15,27,28], was analyzed using linear mixed models. Fitted models included (a) explanatory variable: maternal BC, responses: carbohydrate concentrations and CDI; (b) explanatory variables: carbohydrate concentration and CDI, response: infant BC; (c) explanatory variable: carbohydrate concentration, responses: breastfeeding parameters (24-h milk intake and breastfeeding frequency); and (d) explanatory variables: carbohydrate concentration, breastfeeding frequency, and 24-h milk intake, response: carbohydrate CDI. The fixed effects were infant age (as a categorical variable) included as an interaction with the explanatory variable and a random effect for each participant. The *p*-value associated with the interaction was examined and if it was below 0.05, the results were reported for a model with fixed effects for infant age and the explanatory variable of interest and a random effect for each participant. Where the interaction was not significant, results were reported for the same model fitted without the interaction.

The analyses for systematic differences between measured parameters at different months after birth used linear mixed model with age as effect factor and participant as a random factor. Differences between each month were analyzed using general linear hypothesis tests (Tukey’s all pair comparisons).

Relationships between CDI of carbohydrates measured between 2 and 5, and at 9 and 12 months after birth and changes (Δ) in infant BC and anthropometric parameters between the time points were analyzed using linear regression models.

A false discovery rate (FDR) adjustment [38] was applied to the subgroupings of results to the interaction *p*-value if it was less than 0.05 or to the main effect *p*-value. The adjusted significance levels are reported in the tables or set at the 5% level otherwise. Descriptive statistics are reported as mean ± standard deviation (SD) and range; modeling results as parameters estimates ± standard error (SE). Missing data were dealt with using available case analysis. Statistical analysis and graphics were performed in R 3.1.2.

## 3. Results

### 3.1. Subjects

Participant demographic characteristics; determinants of maternal and infant BC, anthropometrics, and breastfeeding parameters (24-h milk intake, 24-h breastfeeding frequency, self-reported breastfeeding frequency); as well as participant attrition and missing data have been reported in detail previously [15,27,28]. The missing data in this analysis also included carbohydrates concentrations (from 80 expected measurements: TCH (*n* = 12); lactose (*n* = 14); HMO (*n* = 15)) and CDI (from 60 expected measurements: lactose (*n* = 27); TCH and HMO (*n* = 28)); missing data were distributed across the time points (Table 1). In addition to previously reported maternal and infant BC, the current analysis also included maternal and infant FM/FFM ratios (Table 1).

### 3.2. Changes in Body Composition and Human Milk Carbohydrates during First Year of Lactation

Description of the changes in infant and maternal BC and breastfeeding parameters (24-h milk intake, 24-h breastfeeding frequency, self-reported breastfeeding frequency) between 2 and 12 months of lactation have been reported previously [15]. Maternal and infant FM/FFM ratios as well as HM carbohydrates concentrations and CDI over the first year of lactation are detailed in Table 2. 

Maternal FM/FFM decreased across the lactation. Infant FM/FFM measured with BIS initially increased and then decreased at the later months of lactation, while infant FM/FFM measured with either ultrasound 2-skinfolds or ultrasound 4-skinfolds did not differ (Table 2).

Concentrations of all, TCH, lactose and HMO did not differ between 2 and 12 months. CDI of TCH and lactose decreased across the lactation, while HMO CDI did not differ (Table 2).

### 3.3. Maternal Body Composition and Carbohydrates

No associations were seen between maternal BC and concentrations of carbohydrates (data not shown). No associations were seen between maternal BC and CDI of carbohydrates after adjusting for the FDR however, prior to the adjustment higher HMO CDI was associated with higher maternal %FM and FM/FFM between 2 and 5 months, and with lower maternal %FM and FM/FFM at 9 and 12 months (%FM: *p* = 0.014; FM/FFM: *p* = 0.013) (Table A1).

### 3.4. Infant Body Composition and Concentrations of Carbohydrates

**Figure 1:**
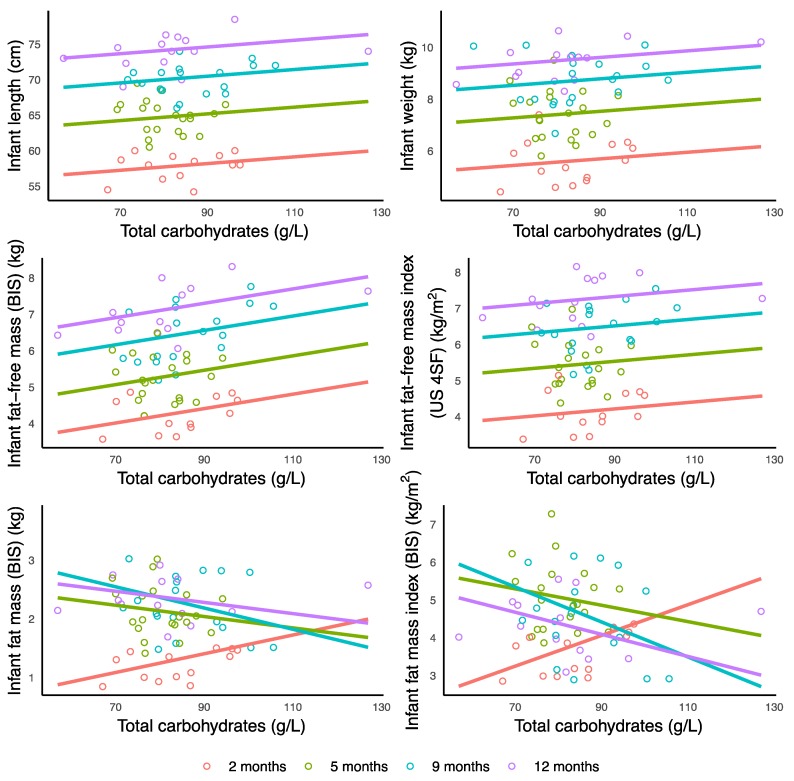
Significant associations between concentration of total carbohydrates and infant anthropometric and body composition measurements. Lines represent linear regression and grouped by the month of lactation. BIS: bioelectrical impedance spectroscopy; US 4SF: ultrasound 4-skinfolds.

Higher TCH concentration was associated with increased infant length (*p* < 0.001), weight (*p* = 0.003), FFM (measured with BIS: *p* < 0.001; ultrasound 4-skinfolds: *p* = 0.020), and FFMI (BIS: *p* = 0.002). Higher TCH concentration was associated with an increase in adiposity measured with BIS (FM: *p* = 0.016; FMI: *p* = 0.001; %FM: *p* = 0.001; FM/FFM: *p* = 0.003) at 2 months and decrease at 5, 9, and 12 months (Table 3, Figure 1).

**Figure 2:**
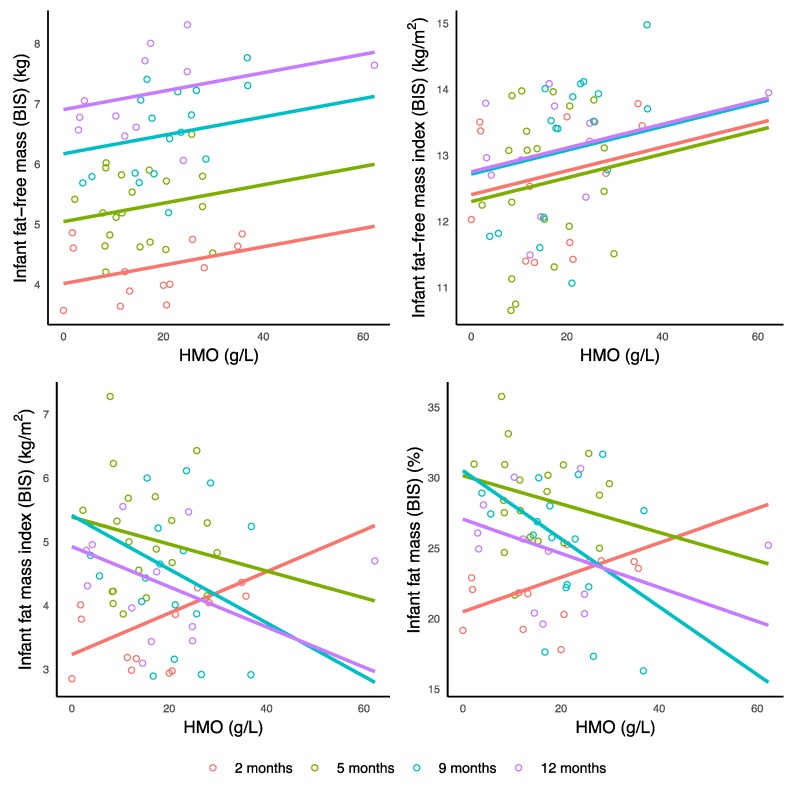
Significant associations between concentration of human milk oligosaccharides (HMO) and infant body composition parameters measured with bioelectrical impedance spectroscopy (BIS). Lines represent linear regression and grouped by the month of lactation.

Higher HMO concentration was associated with increased FFM (BIS: *p* < 0.001) and FFMI (BIS: *p* = 0.008). Higher HMO concentration was associated with an increase in adiposity measured with BIS (FMI: *p* = 0.003; %FM: *p* = 0.005; FM/FFM: *p* = 0.006) at 2 months and decrease at 5, 9 and 12 months (Table 3, Figure 2).

No further associations were seen after adjusting for the FDR (Table 3).

### 3.5. Infant Body Composition and Daily Intakes of Carbohydrates

**Figure 3:**
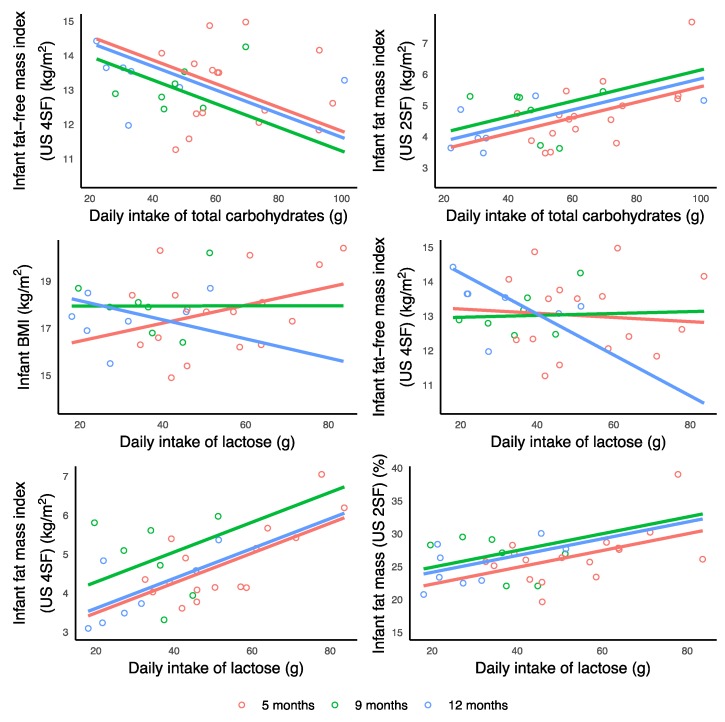
Significant associations between calculated daily intakes of carbohydrates and infant body composition measurements. Lines represent linear regression and grouped by the month of lactation. BMI: body mass index; BIS: US 2SF: ultrasound 2-skinfolds; US 4SF: ultrasound 4-skinfolds.

Higher TCH CDI was associated with increased infant FM (ultrasound 2-skinfolds: *p* = 0.006; ultrasound 4-skinfolds: *p* < 0.001), FMI (ultrasound 2-skinfolds: *p* = 0.003), %FM (ultrasound 2-skinfolds: *p* = 0.005), FM/FFM (ultrasound 2-skinfolds: *p* = 0.004), and decreased FFMI (ultrasound 4-skinfolds: *p* < 0.001); and also with BMI (an increase at 5 months and a decrease at 9 and 12 months, *p* = 0.019), and %FM (*p* = 0.016) and FM/FFM (*p* = 0.007) measured with ultrasound 4-skinfolds (an increase at 5 months and 12 months and a decrease at 9 months for both) (Table 4, Figure 3).

Higher lactose CDI was associated with increased FM (ultrasound 2-skinfolds: *p* = 0.008; ultrasound 4-skinfolds: *p* = 0.004), FMI (ultrasound 2-skinfolds: *p* = 0.005; ultrasound 4-skinfolds: *p* < 0.001), %FM (ultrasound 2-skinfolds: *p* = 0.019; ultrasound 4-skinfolds: *p* = 0.001), FM/FFM (ultrasound 2-skinfolds: *p* = 0.012; ultrasound 4-skinfolds: *p* < 0.001), and also with BMI (an increase at 5 months, no change at 9 months and a decrease at 12 months, *p* = 0.011) and FFMI (ultrasound 4-skinfolds: no change at 5 and 9 months and a decrease at 12 months, *p* = 0.015) (Table 4, Figure 3).

No further associations were found after adjusting for the FDR (Table 4).

### 3.6. Associations between Breastfeeding Parameters and Carbohydrates

No significant associations were found between carbohydrates concentrations and 24-h milk intake, 24-h breastfeeding frequency (meals per 24-h), and self-reported breastfeeding frequency (hours between feeds) (data not shown). Higher concentrations of TCH and HMO were associated with higher CDI of these carbohydrates, respectively (TCH: *p* = 0.009; HMO: *p* = 0.014), while no association was seen between lactose concentration and CDI (Table A2).

A higher 24-h milk intake was associated with higher CDI of all measured carbohydrates (TCH: *p* < 0.001; lactose: *p* < 0.001; HMO: *p* = 0.016). 

**Figure 4:**
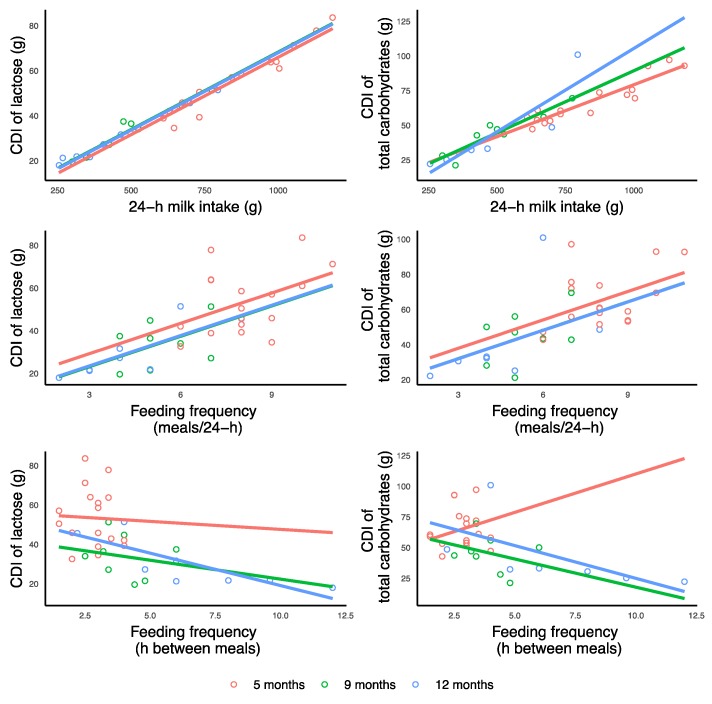
Significant associations between breastfeeding parameters and calculated daily intakes (CDI) of lactose and total carbohydrates measured during 24-h milk production. Lines represent linear regression and grouped by the month of lactation. Where is not seen, the linear regression line for 9 months (green) is under the 12 months line (blue) due to the similar intercept values.

Higher 24-h breastfeeding frequency and lower self-reported breastfeeding frequency were associated with higher CDI of TCH and lactose (24-h breastfeeding frequency: TCH, *p* = 0.009; lactose, *p* < 0.001; self-reported breastfeeding frequency: TCH, *p* < 0.001; lactose, *p* = 0.006) while no associations with HMO CDI were seen (Table A2, Figure 4).

### 3.7. Changes in Infant Body Composition and Calculated Daily Intakes of Carbohydrates

A significant association was found between changes in infant BC (Δ) between the time points and lactose CDI. Higher lactose CDI at 12 months was associated with greater decrease in FFMI (ultrasound 4-skinfolds: *p* = 0.0004) between 5 and 12 months (Table 5). No further significant associations were found at any time points between changes in infant BC (Δ) between the time points and CDI of both TCH and HMO after adjusting for the FDR (Table A3 and Table A4).

## 4. Discussion

**Figure 5:**
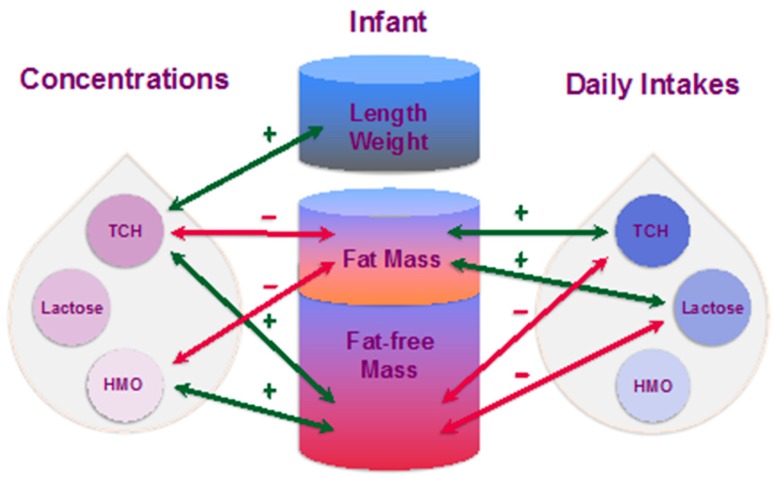
Suggested lactocrine programming of the infant body composition during first 12 months of life. Green arrows indicate positive associations of human milk carbohydrates (concentrations and calculated daily intakes) with infant body composition parameters and red arrows—negative associations. HMO: human milk oligosaccharides; TCH: total carbohydrates.

This proof-of-concept longitudinal study elucidates some of the complex mechanisms by which breastfeeding and HM may affect the development of infant BC and possibly contribute to protection from obesity. Our findings are noteworthy, given the scarcity of data on the effect of HM components, specifically carbohydrates, on infant growth and development. In this study both, concentrations and, more importantly, daily intakes of HM carbohydrates have been found to associate with development of infant BC and are differentially related to infant anthropometry and BC (FM and FFM; Figure 5) throughout the first 12 months of lactation. Moreover, infant breastfeeding frequency was associated with CDI of TCH and lactose, highlighting the important role of breastfeeding in programming of infant appetite regulation and BC in the first year of life.

HM carbohydrates are a major macronutrient which contributes over 40% of energy of the HM [39], predominantly from lactose, and may have greater biological significance compared with other components; thus, it is not surprising that we have established multiple strong associations with infant BC. What is interesting is that the directions of the associations with infant BC were not uniform for the concentration and CDI of the same measured component. This is due to the lack of association between CDI and concentration of lactose, and differences in CDI of TCH and lactose, but not in CDI of HMO, across the time.

Within the normal developmental context of breastfeeding we established that the concentration of lactose, the principal HM carbohydrate, was not related to infant BC or anthropometrics during the first 12 months of lactation (Table 3). Although added dietary sugars have been shown to promote excess adiposity in infants [5], studies of the effect of HM lactose on infant BC are rare. While there has been increased interest in HMO, the importance of lactose for infant development and digestive issues, such as lactose intolerance and malabsorption, is still not well understood. Two recent observational studies found higher HM lactose concentration was related to greater infant weight gain. The first study confirmed a positive association between HM lactose concentration (2–4 weeks lactation) and weight-to-length *z*-score of 6-month-old infants of mothers with excess weight and obesity [40]. The second study also showed that increased HM lactose concentration (4–8 weeks lactation) was related to increased infant weight gain, BMI, and adiposity (skinfolds) at 12 months [22]. Our contrasting results are likely due to the mixed feeding present in both of the previous studies particularly in the first 5 months, and pooling of HM samples over two weeks in one study [22]. On the other hand, our findings are similar to two studies that reported no relationship of lactose concentration with BC of 6-month-old exclusively breastfed infants [23] and infant growth rate (1–6 months) of exclusively breastfed infants (4 or more months) [11], supporting the notion that exclusivity in the first 4–6 months of life may be important. Alternatively, the lack of associations of lactose concentration could be due to its role in maintaining a constant osmotic pressure [10], and thus being one of the least variable HM components [11,14] during the established lactation.

To account for variation in infant 24-h milk intake, which is known to contribute to infant growth [11], we calculated the CDI of carbohydrates and observed that the absence of associations of lactose concentration with infant BC was mediated by daily intakes. CDI of lactose was positively associated with infant adiposity and negatively with lean body mass (Table 4). Furthermore, lactose CDI at 12 months was associated with a decrease in infant FFMI between 5 and 12 months (Table 5). Previously daily lactose intake has not been shown to be related to the growth rate of the 4-month-old breastfed infants [11], however the study sample size was very small (*n* = 6). Another study found contradictory results to ours with higher daily intakes of HM lactose being related to increased weight and lean mass gain but not FM in 3–6 months-old breastfed infants [21]. The hypothesis of the growth regulating effect of lactose is supported by porcine studies that show increased weight gain with administered lactose [41], which has been attributed to the prebiotic effect on gut microbiome resulting in growth of lactobacilli and bifidobacteria, improved intestinal health and better minerals absorption. Further, one in vitro HM study reported that in colonic epithelial cells HM lactose induces the gene (*CAMP*) that encodes an antimicrobial peptide, human cathelicidin LL-37, in both a dose- and time-dependent manner [42]. Furthermore, the *CAMP*-inducing capacity of HM lactose increased with time postpartum, while no *CAMP*-inducing effect was found for lactose in commercial formulas. Authors have speculated that small amount of lactose may not be absorbed and, via the *CAMP*-induction, contribute to infant gut homeostasis, protection against pathogens and regulation of gut microbiome [42], which has been implicated in the development of infant weight gain and obesity [43].

The measured HM concentrations of lactose or TCH in our cohort were not related to maternal adiposity, similar to previous findings in a larger cohort [9]. Given that lactose is important for maintaining a constant osmotic pressure in HM [10], maternal adiposity was not expected to have a significant impact on lactose concentration, although negative associations between the lactose concentration and maternal BMI but not %FM have been reported [44]. In contrast, one longitudinal study found positive association between maternal BMI and TCH concentration [45], however this was in a cohort of women with high milk supply which may have influenced the relationship. 

Lactose accounts for approximately 85% of the total HM carbohydrates [46] and is often considered akin to TCH despite the significant presence of other carbohydrates. HMO is the third most plentiful component of HM, the amount of which often exceeds total amount of protein [17], while monosaccharides (glucose and galactose) are present in HM in very small quantities [36]. We have used the technique that reliably estimates concentrations and carbon content for monosaccharides, disaccharides as well as polysaccharides with very high molecular weight [34,35] and found that higher concentrations of TCH were associated with greater infant length, weight and lean body mass and decreased adiposity (Table 3). However, TCH CDI was associated with increased infant adiposity and reduced lean mass (Table 4), similar to lactose CDI. Comparable to our findings for TCH, higher concentrations of glucose have been reported to associate with higher relative weight and BMI of 1-month-old breastfed infants [24], while concentration of fructose, thought to be present in HM from maternal diet, was positively associated with weight and lean mass in 6-month-old exclusively breastfed infants [23]. Although present in HM in much lower quantities, together with lactose these small carbohydrates may have a synergistic effect, as previously seen in an in vitro study where stimulation of colonic epithelial cells with combinations of various mono- and disaccharides produced enhanced *CAMP* gene expression compared with the individual effects [42], implying potential roles of multiple HM carbohydrates in infant gut homeostasis in addition to HMO. 

Our estimations of HMO also resulted in associations with infant BC. HMO concentration was positively associated with infant lean mass and negatively with adiposity (Table 3), and not HMO CDI (Table 4). Our results are consistent with HMO effects seen in the study of 6-month-old breastfed infants, where concentrations of 16 detected HMO displayed various associations with infant BC, and higher HMO diversity and evenness were associated with lower FM and %FM at 1 month postpartum [20]. Importantly, when examined collectively, the concentration of several HMO in that study explained 33% more of variance in infant adiposity than maternal pre-pregnancy BMI, weight gain during pregnancy or infant sex and age alone. There were discrepancies between HMO concentrations though, with the sum of the 16 individual HMO concentrations (11 g/L at 1 month; 10 g/L at 6 months) [20] being a half of our estimated total HMO concentration at all time points, which could be explained partially by the methodology, with the sulfuric acid–UV method detecting saccharides usually excluded from measurement with HPLC technique, such as acidic oligosaccharides, large oligosaccharides with a degree of polymerization, and small amounts of unidentified saccharides [18]. 

Other studies of concentrations of individual HMO (2’fucosyllactose) have shown no relationships [47]. Further, a study where 2’fucosyllactose and lacto-N-neotetraose were added to formula showed no relationships with infant anthropometrics over the first year of life [48]. Our results indicate that total HMO as opposed to individual HMO are important, and indeed it has been shown that specific bacteria differentially consume specific HMO [49], with a mixture of HMO feeding the whole microbiome. Thus, HMO likely have an indirect effect on BC by enhancing the growth of beneficial gut bacteria and altering the structure or function of the gut microbiome during the critical periods for the development of obesity [43,50]. Directly measuring the wider variety of HMO would be an advantage in the future research.

The recommendations for breastfeeding are to feed on demand. While we have not seen any associations between concentrations of HM carbohydrates and breastfeeding parameters, we found that infants who fed more frequently and had a higher 24-h milk intake consumed higher daily doses (CDI) of lactose and TCH (Table A2). We have previously reported that frequent feeders consume more milk and have lower FFM and higher FM [15], although the order of the relationship is not clear. Further, lactose is also implicated in gastric emptying of breastfed infants, with higher volumes of higher lactose concentration emptying faster compared to lower volumes of lower concentration [51], supported by previous findings in the group that higher lactose concentration is associated with increased breastfeeding frequency [14]. All of these multiple associations culminate in relationships to infant adiposity and highlight the complexity of pathways by which HM components and the dose that the infant receives impact infant development. In contrast, HMO CDI was not associated with breastfeeding frequency in this study, nor were any strong associations previously seen between total HMO concentration/single dose and gastric emptying of breastfed infants [51], indicating the absence of short-term response to total HMO and their target being the small intestine rather than the stomach.

Before the FDR adjustment, we established that HMO CDI was associated with maternal adiposity (%FM and FM/FFM, Table A1), and associations were contrasting at different stages of lactation; during the exclusive breastfeeding period the relationship was positive, but at 9 and 12 months it was negative. This is in agreement with a study that found negative associations between BMI/adiposity and individual HMO concentrations in maternal serum during pregnancy [52], and with two HM studies, which reported positive and negative correlations between maternal BMI and individual HMO [19,53], indicating maternal factors may contribute to total HMO content. It is plausible that relationships of HMO with maternal adiposity may change when mother returns to pre-pregnancy adiposity, or indicate a possible shift in HMO profile later in lactation when volume of milk produced declines. While the total proportion of HMO has been reported to decline during lactation [18], we did not see the decline in total HMO concentrations over the 12 months of lactation. This emphasizes the importance of HMO for the infant development and accounting for the dose of these carbohydrates.

These findings highlight pitfalls in HM research, with HM components concentrations being the major factor measured with respect to various infant outcomes. Pointedly we see none or opposite relationships for these components when analyzing both, raw concentrations and intakes, rising a question if concentration is a dependable measure. When it comes to HM, concentration of component does not necessarily reflect the dose of the component, considering the variability in volumes of HM consumed, duration of exclusive breastfeeding, as well as in breastfeeding patterns and mixed feeding. When examining the nutritional physiology of the breastfed infant CDI may be a more relevant to understanding the development of BC than concentrations.

Our proof-of-concept study concentrated on women that breastfed on demand for 12 months (exclusively to 5 months and without formula use thereafter), and therefore is more indicative of normative development of BC of the breastfed infant as well as lactation. The strengths of this study also include the longitudinal assessment of HM composition and BC of dyads, the broad variation of maternal adiposity and the analysis of TCH along with lactose. The study limitations are the modest number of dyads related to the multiple measurement time points; the small number of 24-h milk productions at the later months of lactation; an estimation of the total HMO concentration, notwithstanding the technical difficulties with accounting for all HMO. We were unable to collect infant dietary intake data after 5 months, yet it is introduction of and duration of use of formula that alters the BC of breastfed infants, not introduction of solids [21]. Our sample was relatively homogenous (primarily Caucasian term healthy fully-breastfed singletons from mothers of higher social-economic status), thus our results may not be transferable to more diverse populations.

## 5. Conclusions

This pilot study has provided the methods and design to identify relationships between the maternal BC, HM carbohydrates, 24-h milk intake, breastfeeding frequency, and infant BC. Our results point to a differential effect of concentrations and daily intakes of HM carbohydrates on development of infant lean mass and adiposity during this early time, indicating a potential to improve infant outcome through continuation of breastfeeding during the first 12 months and beyond, which could promote appropriate developmental programming and impact future metabolic health.

## Figures and Tables

**Table 1:** Fat mass to fat-free mass ratios and concentrations and 24-h intakes of human milk carbohydrates during first 12 months of lactation.

Characteristics	2 Months	5 Months	9 Months	12 Months
	Mean ± SD (Min–Max)	Mean ± SD (Min–Max)	Mean ± SD (Min–Max)	Mean ± SD (Min–Max)
FM/FFM ratios	(*n* = 14)	(*n* = 20)	(*M* = 18/*I* = 17)	(*M* = 17/*I* = 15)
Maternal FM/FFM	0.56 ± 0.14 ^a^ (0.35–0.81)	0.51 ± 0.16 (0.30–0.89)	0.48 ± 0.16 (0.25–0.80)	0.43 ± 0.16 (0.24–0.80)
Infant FM/FFM with US 2SF	0.32 ± 0.04 (0.26–0.61)	0.37 ± 0.09 (0.24–0.64)	0.35 ± 0.08 (0.18–0.53)	0.34 ± 0.06 (0.22–0.45)
Infant FM/FFM with US 4SF	0.33 ± 0.05 (0.24–0.44)	0.36 ± 0.07 (0.26–0.56)	0.34 ± 0.07 (0.21–0.45)	0.31 ± 0.06 (0.21–0.41)
Infant FM/FFM with BIS	0.28 ± 0.04 (0.22–0.34)	0.41 ± 0.07 (0.28–0.56)	0.34 ± 0.08 (0.19–0.46)	0.33 ± 0.06 (0.24–0.44)
**Concentrations**	**(*n* = 15)**	**(*n* = 20)**	**(*n* = 19)**	**(*n* = 14)**
Total carbohydrates (g/L)	86.7 ± 9.2 (67.1–97.5)	80.7 ± 7.9 (69.3–94.1)	87.8 ± 11.1 (60.9–105.6)	88.4 ± 21.2 (56.9–126.9)
Lactose (g/L)	64.5 ± 4.1 (59.1–77.9)	64.3 ± 5.9 (53.5–70.6)	65.3 ± 5.3 (57.6–79.0)	66.9 ± 4.0 (60.1–79.3)
HMO (g/L)	22.3 ± 10.7 (0–35.8)	16.4 ± 9.9 (2.3–29.9)	22.5 ± 9.2 (0–36.9)	21.4 ± 22.3 (3.0–62.2)
**CDI ^b^**	**n/a ^b^**	**(*n* = 17)**	**(*n* = 8)**	**(*n* = 8)**
Total carbohydrates (g)	n/a	63.2 ± 15.0 (42.9–97.2)	44.8 ± 15.2 (21.2–69.6)	40.7 ± 29.8 (22.2–100.9)
Lactose (g)	n/a	51.2 ± 14.5 (32.6–83.6)	34.0 ± 11.0 (19.6–51.3)	28.7 ± 12.1 (18.0–51.4)
HMO (g)	n/a	12.0 ± 6.0 (2.0–21.6)	10.8 ± 5.4 (0–15.7)	12.0 ± 18.5 (1.5–49.5)

^a^ Data are mean ± SD and ranges. ^b^ Daily intakes (CDI) of carbohydrates were calculated between 2 and 5 months (presented at 5 months here) and within 2 weeks of 9 and 12 months. BIS: bioelectrical impedance spectroscopy; CDI: calculated daily intakes; FFM: fat-free mass; FM: fat mass; FM/FFM: fat mass to fat-free mass ratio; HMO: human milk oligosaccharides; I: infants; M: mothers; n/a: not applicable; US 2SF: ultrasound 2-skinfolds; US 4SF: ultrasound 4-skinfolds.

**Table 2:** Differences by infant age/lactation duration within fat mass to fat-free mass ratios and measured human milk carbohydrates ^a^.

Changes in Characteristics between Time Points	Months after Birth						
	5 and 2 Months	9 and 2 Months	12 and 2 Months	9 and 5 Months	12 and 5 Months	12 and 9 Months	Overall *p* Value
**FM/FFM ratios**	**(*n* = 14)**	**(*n* = 12)**	**(*M* = 11/*I* = 10)**	**(*M* = 18/*I* = 17)**	**(*M* = 18/*I* = 17)**	**(*M* = 16/*I* = 13)**	**(*n* = 20)**
Maternal FM/FFM	0.01 ± 0.01 ^b^ (0.98)	−0.02 ± 0.01 (0.63)	−0.04 ± 0.01 ^d^ (0.002)	−0.02 ± 0.01 (0.27)	−0.05 ± 0.01 ^d^ (<0.001)	−0.03 ± 0.01 ^d^ (0.042)	<0.001 ^c,d^
Infant FM/FFM with ultrasound 2-skinfolds	0.03 ± 0.03 (0.69)	0.01 ± 0.03 (0.96)	−0.003 ± 0.03 (1.00)	−0.01 ± 0.02 (0.93)	−0.03 ± 0.02 (0.61)	−0.02 ± 0.03 (0.93)	0.59
Infant FM/FFM with ultrasound 4-skinfolds	0.02 ± 0.02 (0.86)	0.002 ± 0.02 (1.00)	−0.03 ± 0.02 (0.54)	−0.01 ± 0.02 (0.87)	−0.05 ± 0.02 (0.095)	−0.03 ± 0.02 (0.41)	0.16
Infant FM/FFM with BIS	**0.12 ± 0.02** **(<0.001)**	**0.06 ± 0.02** **(0.028)**	0.05 ± 0.02 (0.17)	**−0.06 ± 0.02** **(0.006)**	**−0.07 ± 0.02** **(0.002)**	−0.01 ± 0.02 (0.95)	**<0.001**
**Concentrations**	**(*n* = 15)**	**(*n* = 14)**	**(*n* = 8)**	**(*n* = 19)**	**(*n* = 14)**	**(*n* = 14)**	**(*n* = 20)**
Total carbohydrates (g/L)	−3.0 ± 3.7 (0.86)	1.4 ± 3.8 (0.98)	−1.2 ± 4.1 (0.99)	4.4 ± 3.5 (0.60)	1.8 ± 3.8 (0.97)	−2.6 ± 3.9 (0.91)	0.65
Lactose (g/L)	−1.3 ± 1.8 (0.89)	−1.1 ± 1.8 (0.93)	0.5 ± 1.9 (0.98)	0.2 ± 1.6 (1.00)	1.8 ± 1.8 (0.73)	1.6 ± 1.8 (0.80)	0.70
HMO (g/L)	−2.2 ± 3.9 (0.94)	2.2 ± 3.9 (0.95)	0.7 ± 4.3 (1.00)	4.3 ± 3.6 (0.63)	2.9 ± 3.9 (0.89)	−1.4 ± 4.0 (0.98)	0.69
**CDI**				**(*n* = 7)**	**(*n* = 6)**	**(*n* = 6)**	**(*n* = 9)**
Total carbohydrates (g) ^e^	n/a ^f^	n/a ^f^	n/a ^f^	**−21.0 ± 5.9** **(0.001)**	**−24.7 ± 6.2** **(<0.001)**	−3.7 ± 6.6 (0.84)	**0.003**
Lactose (g) ^e^	n/a	n/a	n/a	**−19.4 ± 4.4** **(<0.001)**	**−23.1 ± 4.4** **(<0.001)**	−3.8 ± 4.8 (0.71)	**<0.001**
HMO (g) ^e^	n/a	n/a	n/a	−1.4 ± 4.0 (0.94)	−1.5 ± 4.2 (0.94)	−0.05 ± 4.9 (1.00)	0.91

^a^ Systematic differences in the measured variables between different months after birth were calculated using general linear hypothesis test (Tukey’s all pair comparisons). ^b^ Data are parameter estimate ± SE of estimate and *p*-value (in parenthesis). ^c^ Overall *p*-value is associated with age as reported in linear mixed model. ^d^ Bold text indicates significant difference (*p* < 0.05) between two time points or overall. ^e^ 24-h milk intake and feeding frequency as meals per 24-h was measured at 24-h milk production and daily intakes (CDI) calculated between 2 and 5 months (*n* = 17) and within 2 weeks of 9 (*n* = 8) and 12 months (*n* = 8). ^f^ Results are not presented for impractical combinations. BIS: bioelectrical impedance spectroscopy; FFM: fat-free mass; FM: fat mass; FM/FFM: fat mass to fat-free mass ratio; HMO: human milk oligosaccharides; I: infants; M: mothers; n/a: not applicable.

**Table 3:** Associations between concentrations of human milk carbohydrates and infant characteristics.

Predictor (Concentration, g/L)	2 Months (*n* = 15)		5 Months (*n* = 20)		9 Months (*n* = 19)		12 Months (*n* = 14)		*P*-Value (*n* = 20)		
	Intercept (SE)	Slope (SE)	Intercept (SE)	Slope (SE)	Intercept (SE)	Slope (SE)	Intercept (SE)	Slope (SE)	Predictor	Infant Age (Months)	Interaction
**Infant Length (cm)**											
TCH	53.90 (1.22) ^a^	0.047 (0.013)	60.90 (1.15)	0.047 (0.013)	66.20 (1.21)	0.047 (0.013)	70.40 (1.17)	0.047 (0.013)	**<0.001 ^b^**	<0.001	0.62 ^c^
Lactose	53.60 (2.31)	0.065 (0.034)	60.50 (2.27)	0.065 (0.034)	66.10 (2.26)	0.065 (0.034)	70.0 (2.27)	0.065 (0.034)	0.047	<0.001	0.093
HMO	57.40 (0.62)	0.031 (0.014)	64.30 (0.55)	0.031 (0.014)	69.70 (0.60)	0.031 (0.014)	73.70 (0.60)	0.031 (0.014)	0.036	<0.001	0.67
**Infant Weight (kg)**											
TCH	4.55 (0.42)	0.013 (0.004)	6.39 (0.40)	0.013 (0.004)	7.65 (0.41)	0.013 (0.004)	8.47 (0.40)	0.013 (0.004)	**0.003**	<0.001	0.54
HMO	5.46 (0.22)	0.009 (0.004)	7.28 (0.21)	0.009 (0.004)	8.54 (0.21)	0.009 (0.004)	9.42 (0.22)	0.009 (0.004)	0.038	<0.001	0.17
**Infant Body Mass Index (kg/m^2^)**											
TCH	11.30 (2.04)	0.063 (0.024)	18.20 (2.38)	−0.007 (0.029)	19.10 (1.83)	−0.016 (0.021)	18.30 (1.29)	−0.013 (0.015)	0.91	<0.001	0.044
HMO	15.70 (0.51)	0.049 (0.020)	17.80 (0.52)	−0.008 (0.026)	17.70 (0.59)	−0.001 (0.023)	17.90 (0.47)	−0.026 (0.016)	0.99	<0.001	0.027
**Infant Fat-free Mass with Bioelectrical Impedance Spectroscopy (kg)**											
TCH	2.63 (0.35)	0.020 (0.004)	3.69 (0.33)	0.020 (0.004)	4.78 (0.35)	0.020 (0.004)	5.53 (0.33)	0.020 (0.004)	**<0.001**	<0.001	0.28
HMO	4.02 (0.17)	0.015 (0.004)	5.05 (0.15)	0.015 (0.004)	6.17 (0.17)	0.015 (0.004)	6.90 (0.17)	0.015 (0.004)	**<0.001**	<0.001	0.069
**Infant Fat-free Mass with Ultrasound 2-skinfolds (kg)**											
TCH	3.41 (0.38)	0.009 (0.004)	4.66 (0.36)	0.009 (0.004)	5.63 (0.38)	0.009 (0.004)	6.32 (0.37)	0.009 (0.004)	0.032	<0.001	0.82
**Infant Fat-free Mass with Ultrasound 4-skinfolds (kg)**											
TCH	3.35 (0.38)	0.010 (0.004)	4.66 (0.36)	0.010 (0.004)	5.64 (0.38)	0.010 (0.004)	6.46 (0.36)	0.010 (0.004)	**0.020**	<0.001	0.86
**Infant Fat-free Mass Index with Bioelectrical Impedance Spectroscopy (kg/m^2^)**											
TCH	10.90 (0.62)	0.022 (0.007)	10.80 (0.58)	0.022 (0.007)	11.20 (0.62)	0.022 (0.007)	11.20 (0.59)	0.022 (0.007)	**0.002**	0.054	0.83
HMO	12.40 (0.28)	0.018 (0.007)	12.30 (0.24)	0.018 (0.007)	12.70 (0.27)	0.018 (0.007)	12.80 (0.27)	0.018 (0.007)	**0.008**	0.030	0.30
**Infant Fat Mass with Bioelectrical Impedance Spectroscopy (kg)**											
TCH	−0.03 (0.71)	0.016 (0.008)	2.91 (0.83)	−0.010 (0.010)	3.81 (0.63)	−0.018 (0.007)	3.14 (0.44)	−0.010 (0.005)	0.051	<0.001	**0.016**
HMO	1.09 (0.17)	0.012 (0.007)	2.26 (0.17)	−0.009 (0.009)	2.56 (0.20)	−0.016 (0.008)	2.57 (0.15)	−0.011 (0.006)	0.11	<0.001	0.039
**Infant Fat Mass with Bioelectrical Impedance Spectroscopy (%)**											
TCH	9.84 (6.56)	0.154 (0.078)	37.20 (7.66)	−0.106 (0.010)	46.80 (5.87)	−0.244 (0.067)	35.60 (4.09)	−0.131 (0.049)	0.002	<0.001	**0.001**
HMO	20.50 (1.54)	0.122 (0.069)	30.10 (1.56)	−0.100 (0.087)	30.50 (1.87)	−0.241 (0.080)	27.00 (1.40)	−0.121 (0.055)	0.020	<0.001	**0.005**
**Infant Fat Mass Index with Bioelectrical Impedance Spectroscopy (kg/m^2^)**											
TCH	0.40 (1.47)	0.041 (0.017)	6.81 (1.72)	−0.022 (0.021)	8.59 (1.32)	−0.046 (0.015)	6.72 (0.92)	−0.029 (0.011)	0.008	<0.001	**0.001**
HMO	3.23 (0.36)	0.033 (0.015)	5.39 (0.36)	−0.021 (0.019)	5.41 (0.43)	−0.042 (0.018)	4.93 (0.32)	−0.032 (0.012)	0.033	<0.001	**0.003**
**Infant Fat Mass to Fat-free Mass Ratio with Bioelectrical Impedance Spectroscopy**											
TCH	0.09 (0.12)	0.003 (0.001)	0.56 (0.14)	−0.002 (0.002)	0.70 (0.10)	−0.004 (0.001)	0.54 (0.07)	−0.003 (0.001)	0.001	<0.001	**0.003**
HMO	0.26 (0.03)	0.002 (0.001)	0.44 (0.03)	−0.002 (0.002)	0.44 (0.03)	−0.004 (0.001)	0.38 (0.03)	−0.002 (0.001)	0.027	<0.001	**0.006**

^a^ Parameter estimate ± SE; effects of predictors taken from linear mixed effects models that accounted for month after birth and an interaction between month after birth and predictor with a random effect per participant; if the interaction is not significant parameter estimates are taken from a model with no interaction. ^b,c^ Results are presented only for interactions or predictors with raw *p*-values < 0.05; after the false discovery rate adjustment the interaction/predictor *p*-values were considered to be significant at <0.032 for total carbohydrates (TCH) concentration (indicated by the bold text), at <0.027 for human milk oligosaccharides (HMO) concentration (indicated by the bold text), and at <0.047 for lactose concentration (none are significant).

**Table 4:** Associations between calculated daily intakes of human milk carbohydrates and infant characteristics.

Predictor (CDI ^d^, g)	Between 2 and 5 Months (*n* = 17)		9 Months (*n* = 8)		12 Months (*n* = 8)		*P*-Value (*n* = 18)		
	Intercept (SE)	Slope (SE)	Intercept (SE)	Slope (SE)	Intercept (SE)	Slope (SE)	Predictor	Infant Age (Months)	Interaction
**Infant Body Mass Index (kg/m^2^)**									
TCH	15.60 (1.30) ^a^	0.033 (0.019)	18.60 (1.47)	−0.013 (0.029)	18.50 (0.72)	−0.018 (0.013)	0.65 ^b^	0.77	**0.019 ^c^**
Lactose	15.70 (1.00)	0.038 (0.018)	17.90 (1.21)	0.0003 (0.032)	19.00 (0.88)	−0.040 (0.025)	0.38	0.59	**0.011**
**Infant Fat-free Mass Index with Ultrasound 4-skinfolds (kg/m^2^)**									
TCH	15.20 (0.56)	−0.034 (0.007)	14.70 (0.47)	−0.034 (0.007)	15.10 (0.45)	−0.034 (0.007)	**<0.001**	0.035	0.37
Lactose	13.30 (0.73)	−0.006 (0.013)	12.90 (0.87)	0.003 (0.023)	15.40 (0.65)	−0.060 (0.018)	0.076	0.13	**0.015**
**Infant Fat Mass with Ultrasound 2-skinfolds (kg)**									
TCH	1.24 (0.29)	0.011 (0.004)	1.82 (0.25)	0.011 (0.004)	1.94 (0.23)	0.011 (0.004)	**0.006**	<0.001	0.29
Lactose	1.23 (0.31)	0.014 (0.006)	1.93 (0.24)	0.014 (0.006)	2.06 (0.21)	0.014 (0.006)	**0.008**	<0.001	0.19
**Infant Fat Mass with Ultrasound 4-skinfolds (kg)**									
TCH	1.09 (0.26)	0.014 (0.004)	1.73 (0.22)	0.014 (0.004)	1.64 (0.20)	0.014 (0.004)	**<0.001**	<0.001	0.16
Lactose	1.16 (0.30)	0.015 (0.005)	1.87 (0.23)	0.015 (0.005)	1.80 (0.21)	0.015 (0.005)	**0.004**	<0.001	0.21
HMO	1.73 (0.14)	0.020 (0.008)	2.13 (0.18)	0.020 (0.008)	1.99 (0.17)	0.020 (0.008)	0.010	0.061	0.77
**Infant Fat Mass with Ultrasound 2-skinfolds (%)**									
TCH	20.10 (2.51)	0.010 (0.036)	21.80 (2.15)	0.010 (0.036)	20.70 (1.98)	0.010 (0.036)	**0.005**	0.64	0.064
Lactose	19.80 (2.80)	0.128 (0.050)	22.30 (2.18)	0.128 (0.050)	21.60 (1.94)	0.128 (0.050)	**0.019**	0.59	0.11
**Infant Fat Mass with Ultrasound 4-skinfolds (%)**									
TCH	14.00 (2.91)	0.193 (0.043)	31.10 (4.47)	−0.083 (0.090)	18.80 (2.05)	0.103 (0.042)	<0.001	0.051	**0.016**
Lactose	18.30 (2.66)	0.156 (0.048)	21.60 (2.10)	0.156 (0.048)	18.70 (1.85)	0.156 (0.048)	**0.001**	0.079	0.069
HMO	24.60 (1.29)	0.168 (0.074)	25.10 (1.64)	0.168 (0.074)	21.30 (1.58)	0.168 (0.074)	0.025	0.095	0.51
**Infant Fat Mass Index with Bioelectrical Impedance Spectroscopy (kg/m^2^)**									
Lactose	3.66 (0.74)	0.027 (0.013)	3.27 (0.58)	0.027 (0.013)	3.55 (0.53)	0.027 (0.013)	0.045	0.62	0.41
HMO	5.37 (0.32)	−0.024 (0.017)	4.51 (0.41)	−0.024 (0.017)	4.64 (0.40)	−0.024 (0.017)	0.049	0.013	0.18
**Infant Fat Mass Index with Ultrasound 2-skinfolds (kg/m^2^)**									
TCH	3.10 (0.61)	0.025 (0.009)	3.64 (0.52)	0.025 (0.009)	3.36 (0.47)	0.025 (0.009)	**0.003**	0.32	0.10
Lactose	3.01 (0.67)	0.032 (0.012)	3.74 (0.51)	0.032 (0.012)	3.47 (0.47)	0.032 (0.012)	**0.005**	0.18	0.27
**Infant Fat Mass Index with Ultrasound 4-skinfolds (kg/m^2^)**									
TCH	1.64 (0.74)	0.048 (0.011)	5.22 (1.12)	−0.007 (0.023)	3.21 (0.52)	0.020 (0.011)	<0.001	0.078	0.038
Lactose	2.72 (0.64)	0.038 (0.012)	3.52 (0.50)	0.038 (0.012)	2.84 (0.46)	0.038 (0.012)	**<0.001**	0.065	0.16
HMO	4.28 (0.32)	0.040 (0.018)	4.43 (0.41)	0.040 (0.018)	3.62 (0.39)	0.040 (0.018)	0.034	0.19	0.68
**Infant Fat Mass to Fat-free Mass Ratio with Bioelectrical Impedance Spectroscopy**									
HMO	0.43 (0.02)	−0.002 (0.001)	0.34 (0.03)	−0.002 (0.001)	0.36 (0.03)	−0.002 (0.001)	0.024	<0.001	0.095
**Infant Fat Mass to Fat-free Mass Ratio with Ultrasound 2-skinfolds**									
TCH	0.23 (0.05)	0.002 (0.001)	0.27 (0.04)	0.002 (0.001)	0.25 (0.04)	0.002 (0.001)	**0.004**	0.68	0.052
Lactose	0.22 (0.06)	0.003 (0.001)	0.28 (0.04)	0.003 (0.001)	0.27 (0.04)	0.003 (0.001)	**0.012**	0.50	0.14
**Infant Fat Mass to Fat-free Mass Ratio with Ultrasound 4-skinfolds**									
TCH	0.12 (0.05)	0.004 (0.001)	0.46 (0.08)	−0.002 (0.002)	0.23 (0.04)	0.002 (0.001)	<0.001	0.058	**0.007**
Lactose	0.21 (0.05)	0.003 (0.001)	0.27 (0.04)	0.003 (0.001)	0.22 (0.04)	0.003 (0.001)	**<0.001**	0.084	0.053
HMO	0.33 (0.02)	0.003 (0.001)	0.34 (0.03)	0.003 (0.001)	0.27 (0.03)	0.003 (0.001)	0.027	0.12	0.45

^a^ Parameter estimate ± SE; effects of predictors taken from linear mixed effects models that accounted for month after birth and an interaction between month after birth and predictor with a random effect for participant; if the interaction is not significant parameter estimates are taken from a model with no interaction. ^b,c^ Results are presented only for interactions or predictors with raw *p*-values < 0.05; after the false discovery rate adjustment the interaction/predictor *p*-values were considered to be significant at <0.038 for calculated daily intake (CDI) of total carbohydrates (TCH) (indicated by the bold text), at <0.045 for lactose CDI (indicated by the bold text), and at <0.010 for human milk oligosaccharides (HMO) CDI (none are significant). ^d^ CDI were measured between 2 and 5 months and within 2 weeks of 9 and 12 months.

**Table 5:** Associations of calculated daily intakes of lactose at the time points with changes in infant body composition between the time points.

Changes in Infant Characteristic (Response)	Months after Birth					
	5 and 2	9 and 2	12 and 2	9 and 5	12 and 5	12 and 9
**Lactose CDI (g) between 2 and 5 months (*n* = 17) ^c^**						
ΔLength (cm)	−0.019 (0.031) ^a^ 0.56 ^b^	0.059 (0.032) 0.11	**0.100 (0.032) ^d^** **0.016**	0.004 (0.026) 0.88	0.021 (0.034) 0.54	0.028 (0.033) 0.41
ΔBMI (kg/m^2^)	**0.049 (0.019)** **0.035**	0.015 (0.030) 0.63	−0.006 (0.038) 0.88	−0.015 (0.018) 0.44	−0.033 (0.023) 0.17	−0.017 (0.013) 0.23
ΔFFM with US 2SF (kg)	−0.013 (0.011) 0.27	**0.024 (0.009)** **0.029**	0.015 (0.014) 0.32	0.003 (0.008) 0.76	0.004 (0.010) 0.66	0.0002 (0.005) 0.96
ΔFFM with US 4SF (kg)	0.003 (0.013) 0.84	**0.043 (0.012)** **0.009**	0.044 (0.019) 0.052	0.005 (0.008) 0.51	0.008 (0.009) 0.41	0.004 (0.005) 0.44
ΔFFM with BIS (kg)	0.009 (0.005) 0.12	0.023 (0.016) 0.19	**0.035 (0.012)** **0.025**	0.004 (0.009) 0.63	0.011 (0.011) 0.32	0.012 (0.010) 0.24
ΔFFMI with US 4SF (kg/m^2^)	0.041 (0.023) 0.12	0.045 (0.026) 0.13	**0.088 (0.035)** **0.044**	0.006 (0.013) 0.64	−0.002 (0.018) 0.91	−0.004 (0.014) 0.77
ΔFM with US 2SF (kg)	**0.029 (0.010)** **0.014**	0.007 (0.010) 0.53	0.014 (0.015) 0.38	−0.004 (0.008) 0.68	−0.005 (0.008) 0.53	0.001 (0.004) 0.87
ΔFMI with US 2SF (kg/m^2^)	**0.057 (0.023)** **0.036**	−0.003 (0.022) 0.91	−0.009 (0.035) 0.80	−0.014 (0.018) 0.46	−0.025 (0.020) 0.23	−0.007 (0.008) 0.40
ΔFMI with BIS (kg/m^2^)	0.026 (0.015) 0.11	0.001 (0.029) 0.98	−0.020 (0.023) 0.42	−0.017 (0.017) 0.33	**−0.045 (0.017)** **0.023**	−0.026 (0.019) 0.19
ΔFM/FFM with US 2SF	**0.007 (0.003)** **0.036**	−0.001 (0.002) 0.60	−0.001 (0.005) 0.93	−0.002 (0.002) 0.35	−0.001 (0.001) 0.67	−0.0004 (0.001) 0.65
ΔFM/FFM with BIS	0.001 (0.001) 0.40	−0.001 (0.003) 0.80	**−0.006 (0.002)** **0.032**	−0.002 (0.001) 0.12	**−0.004 (0.002)** **0.034**	−0.002 (0.002) 0.32
**Lactose CDI (g) at 9 months (*n* = 8) ^c^**						
ΔFM with BIS (kg)	n/a ^e^	0.053 (0.081) 0.58	−0.033 (0.062) 0.65	−0.020 (0.025) 0.46	**−0.037 (0.014)** **0.045**	−0.017 (0.018) 0.39
**Lactose CDI (g) at 12 months (*n* = 8) ^c^**						
ΔFFMI with US 2SF (kg/m^2^)	n/a ^e^	n/a ^e^	−0.082 (0.036) 0.15	n/a ^e^	**−0.097 (0.018)** **0.003**	−0.041 (0.018) 0.072
ΔFFMI with US 4SF (kg/m^2^)	n/a	n/a	−0.075 (0.058) 0.33	n/a	**−0.080 (0.007)** 0.0004 ***	−0.042 (0.026) 0.17
ΔFM with US 2SF (kg)	n/a	n/a	**0.036 (0.010)** **0.032**	n/a	0.019 (0.011) 0.13	0.005 (0.006) 0.48

^a^ Parameter estimate ± SE and ^b^ *P*-values for associations between ^c^ calculated daily intakes (CDI) of lactose (predictor) at given time points and the changes (Δ) in measured variables between different months after birth. ^d^ Results are presented only for variables with at least one significant raw *P*-value (*p* < 0.05, indicated by the bold text); after the false discovery rate adjustment, the predictor *P*-values were considered to be significant at <0.009 for CDI of lactose between 2 and 5 months (none are significant), at <0.045 for CDI at 9 months (none are significant) and at <0.003 for CDI at 12 months (indicated by the bold text and ***). ^e^ Results are not presented for impractical time combinations. BIS: bioelectrical impedance spectroscopy; BMI: body mass index; FFM: fat-free mass; FFMI: fat-free mass index; FM: fat mass; FM/FFM: fat mass to fat-free mass ratio; FMI: fat mass index; n/a: not applicable; US 2SF: ultrasound 2-skinfolds; US 4SF: ultrasound 4-skinfolds.

## Appendix A

**Table A1:** Associations between maternal characteristics and calculated daily intakes of human milk oligosaccharides.

Maternal Predictor	Between 2 and 5 Months (*n* = 17)		9 Months (*n* = 7)		12 Months (*n* = 7)		*p*-Value (*n* = 18)		
	Intercept (SE)	Slope (SE)	Intercept (SE)	Slope (SE)	Intercept (SE)	Slope (SE)	Predictor	Infant Age (Months)	Interaction
**Calculated daily intake of human milk oligosaccharides (g)**									
Fat mass (%)	1.64 (10.50) ^a^	0.322 (0.315)	18.60 (16.00)	−0.209 (0.525)	64.80 (20.10)	−2.01 (0.735)	0.80 ^b^	0.90	**0.014 ^c^**
FM/FFM	6.25 (6.75)	11.80 (12.80)	16.30 (10.80)	−9.10 (23.70)	52.40 (15.40)	−112.0 (40.30)	0.93	0.92	**0.013**

^a^ Parameter estimate ± SE; effects of predictors taken from linear mixed effects models that accounted for month after birth and an interaction between month after birth and predictor with a random effect per participant; if the interaction is not significant parameter estimates are taken from a model with no interaction. ^b,c^ Results are presented only for interactions or predictors with raw *p*-values < 0.05 (indicated by the bold text); after the false discovery rate adjustment, the interaction/predictor *p*-values were considered to be significant at <0.013 for human milk oligosaccharides (none are significant). FF/FFM, fat mass to fat-free mass ratio.

**Table A2:** Associations of daily intakes of human milk carbohydrates with concentrations and breastfeeding parameters.

Predictor	Between 2 and 5 Months (*n* = 17)		9 Months (*n* = 8)		12 Months (*n* = 8)		*P*-Value (*n* = 18)		
	Intercept (SE)	Slope (SE)	Intercept (SE)	Slope (SE)	Intercept (SE)	Slope (SE)	Predictor	Infant Age (Months)	Interaction
**CDI of TCH (g) ^d^**									
Concentration of TCH (g)	21.80 (17.10) ^a^	0.544 (0.207)	−4.05 (19.30)	0.544 (0.207)	−5.47 (18.60)	0.544 (0.207)	**0.009 ^b^**	<0.001	0.50 ^c^
24-h milk intake (g) ^d^	−5.84 (7.05)	0.087 (0.008)	0.92 (5.13)	0.087 (0.008)	0.70 (5.05)	0.087 (0.008)	**<0.001**	0.22	0.059
Feeding frequency (24-h MP) ^d^	21.80 (17.30)	5.39 (2.07)	15.80 (12.30)	5.39 (2.07)	15.80 (11.20)	5.39 (2.07)	**0.009**	0.69	0.13
Feeding frequency (SR) ^d^	77.30 (5.65)	−4.25 (1.35)	60.50 (7.32)	−4.25 (1.35)	68.40 (10.40)	−4.25 (1.35)	**<0.001**	<0.001	0.052
**CDI of lactose (g) ^d^**									
Concentration of lactose (g)	17.80 (24.00)	0.551 (0.366)	−3.56 (25.40)	0.551 (0.366)	−7.77 (25.70)	0.551 (0.366)	0.14	<0.001	0.31
24-h milk intake (g)	−2.80 (2.54)	0.069 (0.003)	−0.50 (1.86)	0.069 (0.003)	−0.78 (1.72)	0.069 (0.003)	**<0.001**	0.33	0.29
Feeding frequency (24-h MP)	15.10 (11.90)	4.74 (1.42)	8.98 (8.45)	4.74 (1.42)	9.29 (7.38)	4.74 (1.42)	**<0.001**	0.51	0.12
Feeding frequency (SR)	62.00 (4.26)	−3.04 (1.04)	45.70 (5.60)	−3.04 (1.04)	49.80 (7.71)	−3.04 (1.04)	**0.006**	<0.001	0.78
**CDI of HMO (g) ^d^**									
Concentration of HMO (g)	2.62 (1.87)	0.612 (0.105)	2.47 (2.96)	0.388 (0.125)	−4.50 (1.91)	0.799 (0.071)	<0.001	0.001	**0.014**
24-h milk intake (g)	10.00 (8.24)	0.003 (0.010)	−0.04 (10.00)	0.022 (0.019)	−16.10 (8.20)	0.057 (0.016)	0.020	0.50	**0.016**
Feeding frequency (24-h MP)	0.42 (9.60)	1.45 (1.15)	3.00 (6.99)	1.45 (1.15)	4.12 (6.32)	1.45 (1.15)	0.22	0.82	0.76
Feeding frequency (SR)	15.30 (3.62)	−1.10 (1.00)	15.10 (5.16)	−1.10 (1.00)	18.10 (7.54)	−1.10 (1.00)	0.29	0.87	0.36

^a^ Parameter estimate ± standard error (SE); effects of predictors taken from linear mixed effects models that accounted for month after birth and an interaction between month after birth and predictor with a random effect per participant; if the interaction is not significant parameter estimates are taken from a model with no interaction. ^b,c^ After the false discovery rate adjustment, the interaction/predictor *p*-values were considered to be significant at <0.05 for all predictors (indicated by the bold text). ^d^ 24-h milk intake and breastfeeding frequency as meals per 24-h were measured at 24-h MP; current breastfeeding frequency was also self-reported by mothers at the session as typical hours between meals; CDI were calculated between 2 and 5 months and within 2 weeks of 9 and 12 months.

**Table A3:** Associations of calculated daily intakes of total carbohydrates at the time points with changes in infant body composition between the time points.

Changes in Infant Characteristic (Response)	Months after Birth					
	5 and 2	9 and 2	12 and 2	9 and 5	12 and 5	12 and 9
**Total carbohydrates CDI (g) between 2 and 5 months (*n* = 17) ^c^**						
ΔLength (cm)	−0.006 (0.026) ^a^ 0.83 ^b^	0.041 (0.028) 0.18	**0.074 (0.026) ^d^** **0.023**	0.003 (0.024) 0.91	0.013 (0.030) 0.67	0.016 (0.030) 0.59
ΔWeight (kg)	**0.018 (0.008)** **0.039**	0.025 (0.012) 0.067	0.025 (0.014) 0.11	0.002 (0.007) 0.81	−0.0002 (0.009) 0.98	−0.001 (0.003) 0.81
ΔBMI (kg/m^2^)	**0.040 (0.016)** **0.037**	0.013 (0.025) 0.61	−0.008 (0.029) 0.79	−0.009 (0.017) 0.59	−0.022 (0.021) 0.30	−0.013 (0.012) 0.31
ΔFFM with US 4SF (kg)	0.013 (0.012) 0.30	**0.034 (0.013)** **0.038**	**0.045 (0.016)** **0.024**	0.004 (0.007) 0.57	0.007 (0.008) 0.39	0.004 (0.004) 0.33
ΔFFM with BIS (kg)	**0.009 (0.004)** **0.045**	0.021 (0.013) 0.16	0.024 (0.010) 0.051	0.003 (0.008) 0.67	0.006 (0.010) 0.55	0.006 (0.009) 0.54
ΔFM with US 2SF (kg)	**0.024 (0.008)** **0.019**	0.007 (0.008) 0.41	0.011 (0.013) 0.42	−0.003 (0.008) 0.72	−0.003 (0.007) 0.65	0.001 (0.003) 0.85
ΔFMI with US 2SF (kg/m^2^)	**0.050 (0.018)** **0.022**	0.001 (0.018) 0.94	−0.004 (0.027) 0.88	−0.013 (0.017) 0.46	−0.020 (0.018) 0.28	−0.006 (0.007) 0.44
ΔFMI with US 4SF (kg/m^2^)	0.006 (0.015) 0.73	−0.043 (0.030) 0.19	−0.064 (0.033) 0.10	−0.014 (0.017) 0.41	−0.029 (0.014) 0.071	**−0.015 (0.006)** **0.031**
ΔFM with US 4SF (%)	−0.037 (0.092) 0.70	−0.247 (0.136) 0.11	**−0.371 (0.139)** **0.032**	−0.058 (0.075) 0.45	−0.115 (0.063) 0.089	−0.060 (0.036) 0.12
ΔFM/FFM with US 4SF	−0.0004 (0.002) 0.83	−0.004 (0.002) 0.12	**−0.007 (0.003)** **0.034**	−0.002 (0.002) 0.13	−0.002 (0.001) 0.26	−0.001 (0.001) 0.31
ΔFM/FFM with BIS	0.001 (0.001) 0.37	−0.001 (0.002) 0.71	**−0.005 (0.002)** **0.040**	−0.002 (0.001) 0.14	−0.003 (0.002) 0.15	−0.001 (0.002) 0.58
**Total carbohydrates CDI (g) at 9 months (*n* = 8) ^c^**						
ΔBMI (kg/m^2^)	n/a ^e^	−0.198 (0.272) 0.54	−0.009 (0.238) 0.97	−0.062 (0.037) 0.16	**−0.081 (0.029)** **0.037**	−0.019 (0.028) 0.52
ΔFFM with US 2SF (kg)	n/a	**0.107 (0.021)** **0.037**	−0.011 (0.032) 0.76	−0.006 (0.017) 0.75	0.002 (0.019) 0.92	−0.008 (0.014) 0.62
ΔFM with BIS (kg)	n/a	−0.029 (0.122) 0.84	−0.095 (0.062) 0.27	−0.022 (0.019) 0.30	**−0.033 (0.010)** **0.018**	−0.011 (0.015) 0.50
ΔFMI with BIS (kg/m^2^)	n/a	−0.114 (0.315) 0.75	−0.181 (0.194) 0.45	−0.051 (0.041) 0.27	**−0.077 (0.028)** **0.040**	−0.027 (0.037) 0.51
**Total carbohydrates CDI (g) at 12 months (*n* = 8) ^c^**						
ΔFFMI with US 2SF (kg/m^2^)	n/a ^e^	n/a ^e^	−0.093 (0.030) 0.092	n/a ^e^	**−0.038 (0.014)** **0.038**	**−0.021 (0.007)** **0.033**
ΔFFMI with US 4SF (kg/m^2^)	n/a	n/a	−0.089 (0.055) 0.25	n/a	**−0.031 (0.009)** **0.029**	−0.022 (0.011) 0.11

^a^ Parameter estimate ± SE of estimate and ^b^ *P*-values for associations between ^c^ calculated daily intakes (CDI) of total carbohydrates (predictor) at given time points and the changes (Δ) in measured variables between different months after birth. ^d^ Results are presented only for variables with at least one significant raw *p*-value (*p* < 0.05, indicated by the bold text); after the FDR adjustment, the predictor *p*-values were considered to be significant at <0.019 for CDI of total carbohydrates between 2 and 5 months, at <0.018 for CDI at 9 months and at <0.029 for CDI at 12 months (none are significant). ^e^ Results are not presented for impractical time combinations. BIS: bioelectrical impedance spectroscopy; BMI: body mass index; FFM: fat-free mass; FFMI: fat-free mass index; FM: fat mass; FM/FFM: fat mass to fat-free mass ratio; FMI: fat mass index; n/a: not applicable; SE: US 2SF: ultrasound 2-skinfolds; US 4SF: ultrasound 4-skinfolds.

**Table A4:** Associations of calculated daily intakes of total carbohydrates at the time points with changes in infant body composition between the time points.

Changes in Infant Characteristic (Response)	Months after Birth					
	5 and 2	9 and 2	12 and 2	9 and 5	12 and 5	12 and 9
**HMO CDI (g) between 2 and 5 months (*n* = 17) ^c^**						
ΔHead circumference (cm)	0.083 (0.054) ^a^ 0.16 ^b^	−0.007 (0.075) 0.93	0.087 (0.063) 0.21	−0.010 (0.030) 0.74	**0.047 (0.018)** **0.023**	0.047 (0.028) 0.12
**HMO CDI (g) at 9 months (*n* = 8) ^c^**						
ΔBMI (kg/m^2^)	n/a ^e^	−0.425 (0.231) 0.21	**−0.401 (0.082) ^d^** **0.040**	−0.247 (0.129) 0.11	**−0.361 (0.053)** **0.001**	−0.114 (0.096) 0.29
ΔFFMI with US 4SF (kg/m^2^)	n/a	−0.130 (0.208) 0.60	−0.211 (0.295) 0.55	−0.024 (0.094) 0.81	−0.185 (0.119) 0.20	**−0.169 (0.058)** **0.032**
ΔFM with BIS ^5^ (kg)	n/a	−0.200 (0.058) 0.075	−0.048 (0.108) 0.70	−0.115 (0.060) 0.12	**−0.110 (0.043)** **0.049**	0.005 (0.059) 0.94
ΔFMI with BIS (kg/m^2^)	n/a	−0.513 (0.176) 0.10	−0.186 (0.257) 0.55	−0.274 (0.119) 0.069	**−0.294 (0.097)** **0.029**	−0.019 (0.143) 0.90
**HMO CDI (g) at 12 months (*n* = 8) ^c^**						
ΔFMI with BIS (kg/m^2^)	n/a ^e^	n/a ^e^	−0.336 (0.327) 0.41	n/a ^e^	**−0.052 (0.017)** **0.032**	−0.037 (0.024) 0.17
ΔFM with BIS (%)	n/a	n/a	−1.488 (1.901) 0.52	n/a	**−0.184 (0.070)** **0.047**	−0.157 (0.124) 0.26
ΔFM/FFM with BIS	n/a	n/a	−0.024 (0.032) 0.53	n/a	**−0.004 (0.001)** **0.021**	−0.003 (0.002) 0.24

^a^ Parameter estimate ± standard errors of estimate and ^b^ *P*-values for associations between ^c^ calculated daily intakes (CDI) of human milk oligosaccharides (predictor) at given time points and the changes (Δ) in measured variables between different months after birth. ^d^ Results are presented only for variables with at least one significant raw *p*-value (*p* < 0.05, indicated by the bold text); after the false discovery rate adjustment, the predictor *p*-values were considered to be significant at <0.023 for CDI of human milk oligosaccharides (HMO) between 2 and 5 months, at <0.001 for CDI at 9 months and at <0.021 for CDI at 12 months (none are significant). ^e^ Results are not presented for impractical time combinations. BIS: bioelectrical impedance spectroscopy; BMI: body mass index; FFM: fat-free mass; FFMI: fat-free mass index; FM: fat mass; FM/FFM: fat mass to fat-free mass ratio; FMI: fat mass index; n/a: not applicable; US 4SF: ultrasound 4-skinfolds.
